# Multiplex Genotyping of Cytokine Gene SNPs Using Fluorescence Bead Array

**DOI:** 10.1371/journal.pone.0118008

**Published:** 2015-02-17

**Authors:** Jung-Pil Jang, In-Cheol Baek, Eun-Jeong Choi, Tai-Gyu Kim

**Affiliations:** 1 Department of Microbiology, College of Medicine, The Catholic University of Korea, Seoul, Korea; 2 Hematopoietic Stem Cell Bank, College of Medicine, The Catholic University of Korea, Seoul, Korea; Chinese Academy of Fishery Sciences, CHINA

## Abstract

Single nucleotide polymorphisms (SNPs) of genes that affect cytokine production and function are known to influence the susceptibility and progression of immune-related conditions such as infection, autoimmune diseases, transplantation, and cancer. We established a multiplex genotyping method to analyze the SNPs of cytokine genes by combining the multiplex PCR and bead array platform. Thirteen cytokine gene regions, including 20 SNPs, were amplified, and allele-specific primer extension was performed in a single tube. High-quality allele-specific primers were selected for signals greater than 1000 median fluorescence intensity (MFI) for positive alleles, and less than 500 MFI for negative alleles. To select and improve the extension primers, modifications for the reverse direction, length or refractory were performed. 24 primers in the forward or reverse direction step and 12 primers in length or refractory modifications were selected and showed high concordance with results by nucleotide sequencing. Among the 13 candidate cytokine genes, the SNPs of 12 cytokine genes, including IL-1α, IL-1R, IL-1RA, IL-1β, IL-2, IL-4, IL-4Rα, IL-6, IL-10, IL-12, TGF-β1, and TNF-α, were successfully defined with the selected allele-specific primers in healthy Korean subjects. Our genotyping system provides a fast and accurate detection for SNPs of multiple cytokine genes to investigate their association with immune-related diseases and transplantation outcomes.

## Introduction

Cytokines produced by a variety of immune cells play an important role in gene activation, as well as growth and differentiation of immune cells in both innate and adaptive immune responses. Cytokines influence the local activation of immune cells, and play a critical role in the regulation of immune and inflammatory responses [1–3]. The cytokine profiles produced by T-helper type 1 (Th1), Th2, Th17, and Treg lymphocytes show distinct patterns. Cytokines produced by TH1 cells include interleukin-2 (IL-2), interferon gamma (IFN-γ) and tumor necrosis factor (TNF), and constitute a pro-inflammatory cytokine profile. On the other hand, cytokines produced by Th2 cells, including IL-4, IL-5 and IL-10, exhibit a predominantly anti-inflammatory cytokine profile [4]. Furthermore, Th17 cells, which produce IL-17, are crucial for defending against fungi and extracellular bacteria. The Th17 cells were shown to play a crucial role in the induction of autoimmune diseases and allergen-specific response [5]. In addition to these effector subsets, the CD4+ Treg, characterized by the forkhead box P3 (Foxp3) protein, suppresses adaptive T-cell responses and prevents autoimmunity [6]. Complex networks of cytokines are involved in dynamic and homeostatic regulation of immune responses and other biological pathways [7].

Several mechanisms contribute to the functionality of single nucleotide polymorphisms (SNPs) in cytokine genes, including amino acid changes (IL-6R, IL-13 and IL-1α), exon skipping (IL-7Rα), proximal promoter variants (IL-1β, IL-RA, IL-2, IL-6, IL-10, IL-12, IL-13, IL-16, TNF, IFN-γ, and transforming growth factor-beta (TGF-β)), distal promoter variants (IL-6 and IL-18) and intronic enhancer variants (IL-8) [8]. Investigation into the associations between cytokine genotypes and susceptibility and prognosis of diseases has implications in disease etiology and potential therapeutic intervention [9,10]. Genotyping technologies include both solid phase of gels and glass slide arrays, as well as homogeneous solution assay formats of mass spectrometry and capillary electrophoresis [11]. Traditionally, genotyping for cytokine SNPs has been conducted using polymerase chain reaction-restriction fragment length polymorphism (PCR-RFLP) (IL-1β -511, IL-1β +3954, IL-2–330, IL-6–174, IL-10–819 and IL-10–592) [12], PCR-SSCP (IL-4 promoter-590, IL-4 receptor α and IL-10–1082) [13] and PCR-SSP (IL-6–174, IL-10–1082, -819 and-592) [14,15]. Furthermore, multiplexing methodologies have the advantage of simultaneous detection of multiple nucleic acid sequences in a single reaction, which reduces the time, labor, and costs that are required, as compared to single-reaction-based detection methods [11]. As a high-throughput technique, pyrosequencing (IL-1β −31, IL-6 −174, −572, −597, IL-10 and IL-12β 3′+1158) [16] and microarrays (IL-6 −596, −572, −174, TNF-α −308, −238, IL-1β −511 and −31) [17] have been developed.

Microsphere-based suspension array has been used as a platform for high-throughput nucleic acid detection in various fields [11,18–24]. Advantages of the suspension array technology include fast data acquisition, excellent sensitivity and specificity, and capacity for multiplexed analysis [11].

The present study demonstrated the improvement of allele specific extension primers by several modification strategies and validated a high-throughput genotyping assay using a combine platform of multiplex PCR and Luminex xTAG to examine several cytokine SNPs. Such assay systems could be implemented in small pilot studies or large-scale population studies to investigate the association between multiple cytokine SNPs and immune-related diseases.

## Materials and Methods

### 1. DNA samples

A total of 152 healthy Korean subjects were included as controls in this study, comprising staff and students from the College of Medicine at the Catholic University of Korea. DNA was isolated from peripheral blood using the AccuPrep Genomic DNA Extraction kit (Bioneer Corporation, Daejeon, Korea), following the manufacturer’s instructions. DNA concentration was adjusted to 100 ng/μl and used as PCR templates for genotyping.

### 2. Ethics statement

This study was approved by the Institutional Review Board (IRB) of the Catholic University of Korea (IRB Number: MC13SISI0126). Written informed consent was obtained from all participates involved in this study.

### 3. Quality control panel for genotyping and primer design for multiplex PCR

We performed a study on 20 SNPs for 13 cytokine genes in which genotyping results from multiplex PCR were confirmed, with results from the quality control genotyping panel acquired from sequencing. SNP-specific primer for multiplex PCR was designed following the methodology described by Kaarel et al [25]. Each specific primer was designed based on an optimal melting temperature between 56°C and 60°C. The melting temperature was calculated using the the Oligo Calculator (http://mbcf.dfci.harvard.edu/docs/oligocalc.html). In addition, each specific primer was designed to cover one span between the forward and reverse primers. However, the span of the TGF-β1 specific primer was designed differently at 46 bp. First, the PCR primer was added to a universal sequence (5′-GATCAGGCGTCTGTCGTGCTC-3′) at the 5′ end of each specific primer (Table 1).

**Table 1:** List of primer sequences for multiplex PCR amplifications.

Group	Gene	SNP Position	rs Number	Direction	Sequence (5′- 3′)	Span(bp)*	Tm	Length	Amplicon Size (bp)
**Proinflammatory**	IL-1α	-889	rs1800587	Forward	GAGAAATTCTTTAATAATAGTAACCAGGCAACA	1	57	33	105
				Reverse	CATGGATTTTTACATATGAGCCTTCAATG		56	29	
	IL-1β	+3962	rs1143634	Forward	TGCTCCACATTTCAGAACCTATCTTCTT	1	57	28	96
				Reverse	CATAAGCCTCGTTATCCCATGTGTC		58	25	
		-511	rs16944	Forward	TCCTGCAATTGACAGAGAGCTCC	1	57	23	87
				Reverse	TTGGGTGCTGTTCTCTGCCTC		56	21	
	IL-1R	+1970	rs2234650	Forward	AGGGGGAAAGCCCGAGGGAG	1	60	20	84
				Reverse	GGCGCAGTGGTCGAGTCTGCA		60	21	
	IL-1RA	+11100	rs315952	Forward	CGCCTTCATCCGCTCAGACAG	1	58	21	85
				Reverse	CTCAAAACTGGTGGTGGGGCC		58	21	
	IL-6	-174	rs1800795	Forward	ACTTTTCCCCCTAGTTGTGTCTTGC	1	58	25	94
				Reverse	TTGTGCAATGTGACGTCCTTTAGCAT		56	26	
**Th1**	IL-2	+166	rs2069763	Forward	ACACAGCTACAACTGGAGCATTTACT	1	56	26	102
				Reverse	ATTAATTCCATTCAAAATCATCTGTAAATCCAG		56	33	
		-330	rs2069762	Forward	CAATATGCTATTCACATGTTCAGTGTAGTTTTA	1	57	33	109
				Reverse	GATACAAAAGTAACTCAGAAAATTTTCTTTGTC		56	33	
	IL-12	-1188	rs3212227	Forward	TCACAATGATATCTTTGCTGTATTTGTATAGTT	1	56	33	104
				Reverse	CTGATTGTTTCAATGAGCATTTAGCATC		56	28	
	IFN-γ	5644	rs2430561	Forward	CACAATTGATTTTATTCTTACAACACAAAATCAAATC	1	57	37	106
				Reverse	AGTGTGTGTGTGTGTGTGTGTGTGTG		60	26	
	TNF-α	-308	rs1800629	Forward	GAGGCAATAGGTTTTGAGGGGCATG	1	59	25	88
				Reverse	CTGGAGGCTGAACCCCGTCC		60	20	
		-238	rs361525	Forward	CAGAAGACCCCCCTCGGAATC	1	58	21	86
				Reverse	ACACTCCCCATCCTCCCTGCTC		60	22	
**Th2**	IL-4	-1098	rs2243248	Forward	AGGGCTGATTTGTAAGTTGGTAAGAC	1	56	26	96
				Reverse	CCTCAGCTAATTAGGAAAAAGAGCTAC		57	27	
		-590	rs2243250	Forward	CAAAACACCTAAACTTGGGAGAACATTGT	1	57	29	93
				Reverse	TCTCCTACCCCAGCACTGGGG		60	21	
	IL-4Rα	1902	rs1801275	Forward	GGCCCCCACCAGTGGCTATC	1	60	20	85
				Reverse	CTCCACCGCATGTACAAACTCC		57	22	
	IL-10	-1082	rs1800896	Forward	ACAACACTACTAAGGCTTCTTTGGGA	1	56	26	92
				Reverse	CTCTTACCTATCCCTACTTCCCC		57	23	
		-819	rs1800871	Forward	GTGTACCCTTGTACAGGTGATGTAA	1	56	25	92
				Reverse	GTGAGCAAACTGAGGCACAGAGAT		57	24	
		-592	rs1800872	Forward	ACATCCTGTGACCCCGCCTGT	1	58	21	88
				Reverse	TTTCCAGAGACTGGCTTCCTACAG		57	24	
**Immune modulatory**	TGF-β1	codon 10	rs1800470	Forward	CGGGCTGCGGCTGCTGC	46	59	17	123
		codon 25	rs1800471	Reverse	GATAGTCCCGCGGCCGGC		59	18	
	Universal Primer				GATCAGGCGTCTGTCGTGCTC		58	21	

* span: The distance between a forward primer and reverse primer on target sequence.

IL: interleukin, IFN: interferon, TNF: tumor necrosis factor, TGF: transforming growth factor, R: receptor, RA: receptor antagonist

Universal primer: The universal sequences were not matched with any genomic DNA sequences. All the primers include a universal sequence at individual 5′-end.

### 4. Multiplex PCR

Genomic DNA (100 ng/μl in distilled water) was denatured at 98°C, and then cooled at 4°C prior to PCR. The first PCR included 100 ng of genomic DNA, 300 nM of each SNP specific primer, PCR buffer (50 mM Tris base pH 8.7, 20 mM ammonium sulfate, 2 mM MgCl_2_, 0.1 mg/ml BSA), 0.2 mM of each dNTP, distilled H_2_O and 1.25 U of Taq DNA polymerase (Genelabs, Seongnam, Korea) in a total volume of 15 μl. Amplification was performed in a GeneAmp PCR System 9700 thermocycler (Applied Biosystems, Foster City, CA, USA) using the following conditions: 1 cycle at 98°C for 45 s; 35 cycles of denaturation at 95°C for 20 s, annealing at 60°C for 2 min, extension at 72°C for 20 s. The duration between the annealing and extension steps in the GeneAmp PCR System 9700 thermocycler was set at a 3% ramp speed. The length of the amplified products was confirmed by electrophoresis on a 2% agarose gel.

The next PCR was carried out with the universal primer. The second PCR step included 5 μl of the first PCR product, 40 nM of the universal primer (5′-GATCAGGCGTCTGTCGTGCTC-3′), PCR buffer (75 mM Tis HCl (pH9.0), 50 mM KCl, 20 mM (NH_4_)_2_SO_4_, 2 mM MgCl_2_), 0.3 mM of each dNTP, distilled H_2_O and 5 U Taq DNA polymerase (Biotools B&M Labs, Madrid, Spain) in a total volume of 50 μl. Amplification was performed in a GeneAmp PCR System 9700 thermocycler (Applied Biosystems, Foster City, CA, USA) using the following conditions: 1 cycle at 95°C for 10 min; 35 cycles of denaturation at 95°C for 30 s, annealing at 54°C for 30 s, extension at 72°C for 5 s. Amplified product size was confirmed by electrophoresis on a 2% agarose gel.

### 5. Allele-specific primer extension (ASPE)

The products from the second PCR step were treated with shrimp alkaline phosphatase (SAP) to eliminate the activity of deoxynucleotides. The final volume of the phase enzyme treatment reaction was 20 μl, and contained 17 μl of the second PCR product, SAP reaction buffer (20 mM Tris-HCl (pH 8.0), 10 mM MgCl_2_) and 0.25 U SAP (both from USB, Ohio, USA). The reactions were incubated at 37°C for 30 min, and then inactivated by heating at 95°C for 10 min.

Multiplex allele-specific primer extension reactions were carried out in a total volume of 20 μl. The reaction components were 10 μl of enzyme-treated multiplex PCR products, 1 pM of each tag-allele specific extension primers, PCR buffer (75 mM Tis HCl (pH 9.0), 50 mM KCl, 20 mM (NH_4_)_2_SO_4_, 2 mM MgCl_2_), 400 μM biotin deoxycytidine triphosphate (Biotin-dCTP), 100 μM deoxyadnosine triphosphate (dATP), deoxyguanosine triphosphate (dGTP), deoxythymidine triphosphate (dTTP) mix, distilled H_2_O and 0.5 U Taq DNA polymerase (Biotools B&M Labs, Madrid, Spain). The reactions were carried out using a GeneAmp PCR System 9700 thermocycler (Applied Biosystems, Foster City, CA, USA) under the following conditions: 1 cycle at 95°C for 5 min; 5 cycles of denaturation at 95°C for 10 s; and annealing and extension at 60°C for 5 min.

### 6. Hybridization

For hybridization, 25 μl of 2X Tm hybridization buffer (0.4 M NaCl, 0.2 M Tris (pH 8.0),

0.16% Triton X-100) containing 2500 of each tag-coupled microsphere set was added to 20 μl of the ASPE reaction products and 5 μl distilled H_2_O to achieve a final volume of 50 μl. The mixtures were denatured by heating at 95°C for 90 s and then incubated at 37°C for 1 h. The hybridized microspheres were washed twice with 75 μl of 1X Tm hybridization buffer (0.2 M NaCl, 0.1 M Tris (pH 8.0), 0.08% Triton X-100) followed by centrifugation at 8000 g for 2 min. The supernatant was removed, and 75 μl of 1X Tm hybridization buffer containing 2 μg/mL streptavidin-R-phycoerythrin was added to the microsphere-hybridized ASPE reaction products. The reaction was incubated at 37°C for 15 min. Microsphere fluorescence was measured using a Luminex 200 analyzer (Luminex Corporation, Austin, TX, USA) at 37°C and analyzed with the associated software. The phycoerythrin (PE) median fluorescence intensities (MFI) were determined for each bead, and MFI ratios were calculated for bead pairs specific for the alternate alleles of each cytokine.

### 7. Data Analysis

The allele frequencies were determined using Microsoft Office Excel. Hardy-Weinberg equilibrium (HWE) in controls was analyzed for each SNP using SNPStats (http://bioinfo.iconcologia.net/snpstats/start.htm).

## Results

### 1. Two-step multiplex PCR for 20 SNPs of 13 cytokines

To determine simultaneously the 20 SNPs of 13 cytokine genes in a single tube, the target genes were amplified by nested PCR. These cytokine SNPs have been studied during the Cytokine Component of the 13^th^ International Histocompatibility Workshop. These cytokine gens can be grouped into pro-inflammatory cytokines (IL-1, IL-1R and IL-6), Th1 cytokines (IL-2, IL-12, INF-γ and TNF-α), Th2 cytokines (IL-4, IL-4Rα and IL-10) and Immunemodulatory (TGF-β).

**Fig 1:**
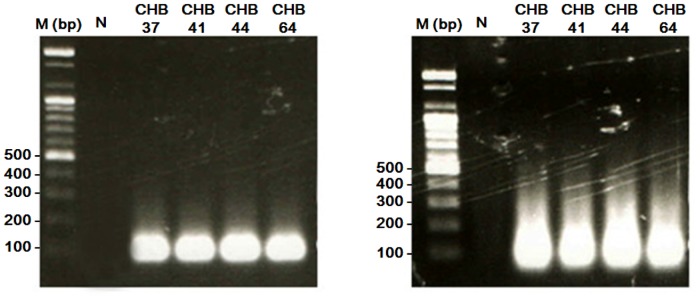
Agarose gel electrophoresis images of cytokine genes amplified by multiplex PCR. (A) The first polymerase chain reaction with 3% ramp speed was amplified by specific sequences, and the size of the amplified products were approximately 100 bp. (B) Products from the second polymerase chain reaction were amplified by the universal sequences and the size of the amplified products were approximately 100 bp as well. The DNA marker (M) is a commercial 100 bp ladder.

Nineteen pairs of primer were used for the first PCR reaction, and the nucleotide sequences of the universal primer used in the second PCR were designed in the absence of the genomic sequence. This two-step multiplex PCR was optimized for several parameters, including primer concentration, number of PCR cycles, and annealing temperature, as described in Materials and Methods. Optimal conditions yielded PCR products of equal sizes, as shown in Fig. 1. The first PCR primers and the universal primer were verified for the amplification of the target genes without cross-hybridization from the SNP genotyping results using fluorescence bead arrays.

### 2. Quality control panel

A total of 20 SNPs belonging to 13 cytokine genes were genotyped in 152 healthy Korean subjects by direct nucleotide sequencing, using the sequencing primers. To validate the performance characteristics of fluorescence bead array and determine the specificity of extension primers, 12 genomic DNA samples representing SNPs in Koreans subjects were selected as the quality control panel. SNP genotypes of 13 cytokines in 12 samples are shown in Table 2. Among the 20 SNP regions, 12 regions showed two homozygote and heterozygote genotypes and 6 regions displayed one homozygote and heterozygote genotypes in Koreans. However, TGF-β1 (codon 25) and IL-6 (-174) regions showed homozygote genotype of only one allele. Therefore, the specificity of the 30 extension primers in 18 SNP regions could be extensively tested using this quality control panel.

**Table 2:** List of quality control panel genotypes by sequencing.

	Proinflammatory						Th1						Th2						Immune modulatory	
Sample	IL-1α(-889)	IL-1(+3962)	IL-1β(-511)	IL-1R(+1970)	IL-1RA(+11100)	IL-6(-174)	IL-2(+166)	IL-2(-330)	IL-12(-1188)	INF-γ(5644)	TNF-α(-308)	TNF-α(-238)	IL-4(-1098)	IL-4 (-590)	IL-4Rα(1902)	IL-10(-1082)	IL-10(-819)	IL-10(-592)	TGF-β1(codon 10)	TGF-β1(codon 25)
**CHB-37**	CC	CC	CT	CC	CT	GG	GG	GG	CA	AA	GA	GG	GT	TT	CT	AA	CT	CA	TT	GG
**CHB-41**	CC	CC	TT	CC	CT	GG	TT	GT	CC	AT	GA	GG	TT	TT	TT	AA	TT	AA	TT	GG
**CHB-44**	CT	CC	CT	CT	TT	GG	GT	TT	AA	AA	GG	GG	TT	TT	CT	AA	TT	AA	TT	GG
**CHB-44**	CC	CC	TT	TT	TT	GG	TT	TT	CA	AA	GG	GG	TT	TT	TT	AA	TT	AA	CT	GG
**CHB-64**	CT	CT	CC	CT	CC	GG	GT	GT	CC	AA	GA	GG	GT	TT	TT	GA	CT	CA	TT	GG
**CHB-72**	CC	CT	CT	CC	CT	GG	GG	GG	CC	AA	GA	GG	TT	TT	CT	GA	CT	CA	TT	GG
**CHB-82**	CC	CC	TT	CC	CT	GG	GT	GT	CA	TT	GA	GG	TT	TT	CT	AA	CC	CC	TT	GG
**CHB-85**	CC	CT	TT	CT	CC	GG	GT	GT	AA	AA	GA	GG	TT	CT	CT	AA	CT	AA	CT	GG
**CHB-105**	CT	CT	CT	CT	CT	GG	GT	GT	CA	AT	GG	GG	TT	CT	CT	AA	TT	AA	CT	GG
**CHB-107**	CC	CC	CC	CT	CT	GG	TT	TT	CA	AA	GA	GG	TT	TT	CC	AA	TT	CC	CT	GG
**CHB-133**	CC	CC	TT	CT	CC	GG	TT	TT	CC	AA	GA	GG	TT	TT	TT	AA	TT	AA	TT	GG
**CHB-157**	CC	CC	CC	CT	CC	GG	GT	TT	CA	AA	GA	GA	TT	CC	TT	AA	TT	AA	CT	GG

IL: interleukin, IFN: interferon, TNF: tumor necrosis factor, TGF: transforming growth factor, R: receptor, RA: receptor antagonist.

### 3. Selection of high-quality extension primers

**Fig 2:**
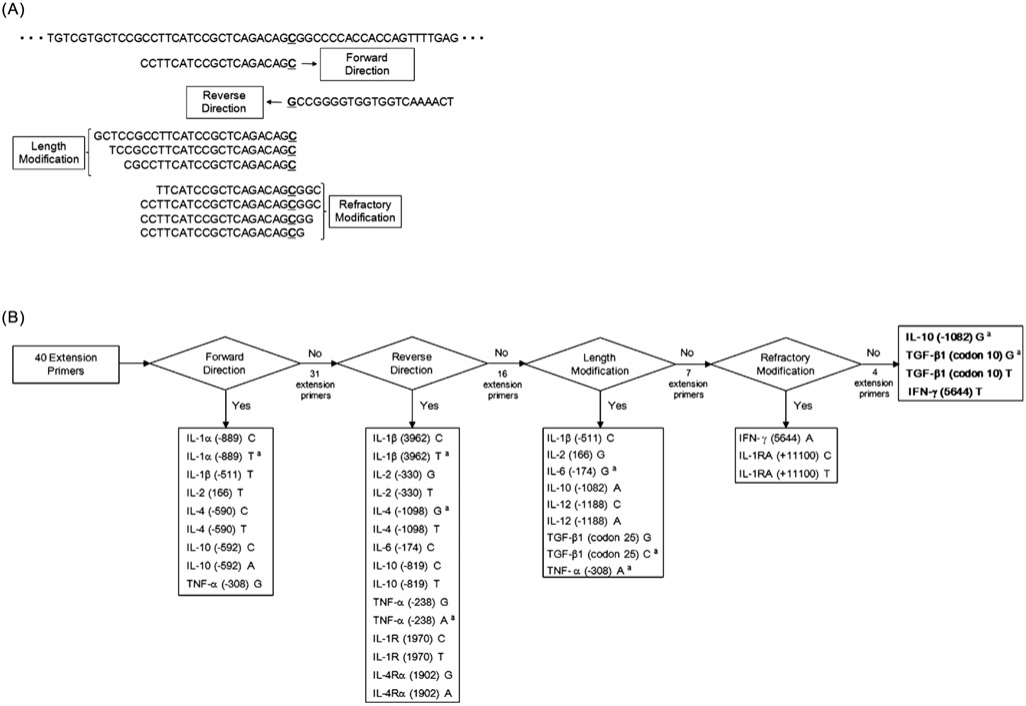
Principle of design and strategies for optimal selection on extension primers. (A) The design principle for the extension primers of IL-1RA (+11100) sequences. (B) The system for primer selection is divided into four major steps. In the forward direction step, nine of the forty primers were selected. The reverse direction was successful in fifteen of the thirty-one primers. The length modification step improved primer quality by adding or removing sequences at the 5′ end of each primer. In this step, nine of the sixteen primers were selected. In the last stage, the primers were improved by moving 1, 2 or 3 bp upstream of the SNP region at the allele-specific 3′ end. Three of the five primers were selected by the refractory system. Finally, 36 of the 40 primers were selected, but four primers (IL-10 (-1082) G, TGFβ1 (codon 10) G, T, IFN-γ (5644) T) were not selected. ^a^: Only the specificity of the primer was confirmed by QC panel because positive quality is not available.

After multiplex PCR amplification of the quality control panel, the fluorescence signals of allele-specific extension primers in bead arrays were measured. In this study, allele-specific primers showing signals higher than 1000 MFI in the positive allele and lower than 500 MFI in the negative allele were determined as high quality. All primers presented in Table 1 were designed in the forward direction with melting temperatures within the range of 56°C to 60°C. None of the high-quality forward direction primers were improved in the reverse direction, length and for refractory extension. Reverse modification primers were designed in the 3′-to-5′ direction instead of the standard 5′-to-3′ direction. Length modification was performed by adding or removing nucleotide sequences at the 5′ end of forward or reverse primers. Refractory primers were modified by moving the 3′ end of the allele specific primer at 1 to 3 bases upstream of SNP region (Fig. 2A). The process of primer selection is presented in Fig. 2B.

The 9 forward direction extension primers (IL-1α (-889) C, IL-1α (-889) T, IL-1β (-511) T, IL-2 (166) T, IL-4 (-590) C, IL-4 (-590) T, IL-10 (-592) C, IL-10 (-592) A and TNF-α (-308) G) were selected as high quality among the first 40 forward direction primers designed. These primers were between 24 to 37 bases in length, with melting temperatures of 56.6 ± 0.7°C, and GC contents of 45.4 ± 11.5% (Table 3). Nineteen of the 31 primers (IL-1β (-511) C, IL-2 (+166) G, IL-4 (-1098) T, IL-6 (-174) G, IL-6 (-174) C, IL-10 (-1082) G, IL-10 (-819) C, IL-10 (-819) T, IL-12 (-1188) C, IL-12 (-1188) A, TGF-β1 (codon 25) G, TGF-β1 (codon 25) C, TNF-α (-308) A, TNF-α (-238) G, TNF-α (-238) A, IFN-γ (5644) A, IFN-γ (5644) T, IL-4Rα (1902) C and IL-4Rα (1902) T) showed false positive results, and the remaining 12 primers showed false negative. The 31 primers of low quality were 18–37 bases in length, with melting temperatures of 57.2 ± 1.6°C, and GC contents of 51.4 ± 17.2%.

**Table 3:** The final list of improved extension primers in the high-quality primer selection system.

Gene	SNP Position	Direction	Length (bp)	Refractory	SNP	Primer Sequence	Tm (°C)	GC (%)
**Forward Direction**								
IL-1α	-889	Forward			C	GAAATTCTTTAATAATAGTAACCAGGCAACA**C**	57	31
IL-1α	-889	Forward			T	AGAAATTCTTTAATAATAGTAACCAGGCAACA**T**	56	27
IL-1β	-511	Forward			T	CCTGCAATTGACAGAGAGCTCC**T**	57	52
IL-2	+166	Forward			T	CACAGCTACAACTGGAGCATTTACT**T**	56	42
IL-4	-590	Forward			C	CACCTAAACTTGGGAGAACATTGT**C**	56	44
IL-4	-590	Forward			T	AAACACCTAAACTTGGGAGAACATTGT**T**	56	36
IL-10	-592	Forward			C	ATCCTGTGACCCCGCCTGT**C**	58	65
IL-10	-592	Forward			A	ATCCTGTGACCCCGCCTGT**A**	56	60
TNF-α	-308	Forward			G	GCAATAGGTTTTGAGGGGCATG**G**	57	52
**Reverse Direction**								
IL-1β	+3962	Reverse			C	TAAGCCTCGTTATCCCATGTGTC**G**	57	50
IL-1β	+3962	Reverse			T	TAAGCCTCGTTATCCCATGTGTC**A**	56	46
IL-2	-330	Reverse			G	ATACAAAAGTAACTCAGAAAATTTTCTTTGTC**C**	56	27
IL-2	-330	Reverse			T	GATACAAAAGTAACTCAGAAAATTTTCTTTGTC**A**	56	26
IL-4	-1098	Reverse			G	CTCAGCTAATTAGGAAAAAGAGCTAC**C**	57	41
IL-4	-1098	Reverse			T	CCTCAGCTAATTAGGAAAAAGAGCTAC**A**	57	39
IL-6	-174	Reverse			C	TGCAATGTGACGTCCTTTAGCAT**G**	56	46
IL-10	-819	Reverse			C	GAGCAAACTGAGGCACAGAGAT**G**	57	52
IL-10	-819	Reverse			T	TGAGCAAACTGAGGCACAGAGAT**A**	56	46
TNF-α	-238	Reverse			G	CTCCCCATCCTCCCTGCTC**C**	60	70
TNF-α	-238	Reverse			A	CTCCCCATCCTCCCTGCTC**T**	58	65
IL-1R	+1970	Reverse			C	CGCAGTGGTCGAGTCTGCA**G**	58	65
IL-1R	+1970	Reverse			T	GCGCAGTGGTCGAGTCTGCA**A**	58	62
IL-4Rα	1902	Reverse			G	TCCACCGCATGTACAAACTCC**C**	57	55
IL-4Rα	1902	Reverse			A	CTCCACCGCATGTACAAACTCC**T**	57	52
**Length Modification**								
IL-1β	-511	Forward	24		C	TCCTGCAATTGACAGAGAGCTCC**C**	59	54
IL-2	+166	Forward	23		G	AGCTACAACTGGAGCATTTACT**G**	53	43
IL-6	-174	Reverse	22		G	CAATGTGACGTCCTTTAGCAT**C**	53	45
IL-10	-1082	Forward	24		A	ACACTACTAAGGCTTCTTTGGGA**A**	56	42
IL-12	-1188	Reverse	24		C	TGTTTCAATGAGCATTTAGCATC**G**	52	38
IL-12	-1188	Reverse	23		A	GTTTCAATGAGCATTTAGCATC**T**	50	35
TGF-β1	codon 25	Forward	25		G	GCTCACTGGTGCTGACGCCTGGCC**G**	68	72
TGF-β1	codon 25	Forward	25		C	GCTCACTGGTGCTGACGCCTGGCC**C**	68	72
TNF-α	-308	Forward	22		A	CAATAGGTTTTGAGGGGCATG**A**	53	45
**Refractory Mdification**								
IFN-γ	5644	Reverse		21(m-3)	A	GTGTGTGTGTGTGTGTG**T**GAT	52	48
IL-1RA	+11100	Forward		21(m-3)	C	TTCATCCGCTCAGACAG**C**GGC	58	62
IL-1RA	+11100	Forward		21(m-1)	T	GCCTTCATCCGCTCAGACAG**T**G	59	59

IL: interleukin, IFN: interferon, TNF: tumor necrosis factor, TGF: transforming growth factor, R: receptor, RA: receptor antagonist

The SNP site in each primer is underlined.

Each extension primer used in this study has a unique “tag” sequence in the 5′ region

(An “anti-tag” that is a complementary “tag” immobilizes on the microspheres).

To improve the 31 failed forward primers, primers in the reverse direction were generated and tested. Fifteen reverse direction primers (IL-1β (+3962) C, IL-1β (+3962) T, IL-2 (-330) G, IL-2 (-330) T, IL-4 (-1098) G, IL-4 (-1098) T, IL-6 (-174) C, IL-10 (-819) C, IL-10 (-819) T, TNF-α (-238) G, TNF-α (-238) A, IL-1R (+1970) C, IL-1R (+1970) T, IL-4Rα (1902) G and IL-4Rα (1902) A) showed high quality.

**Fig 3:**
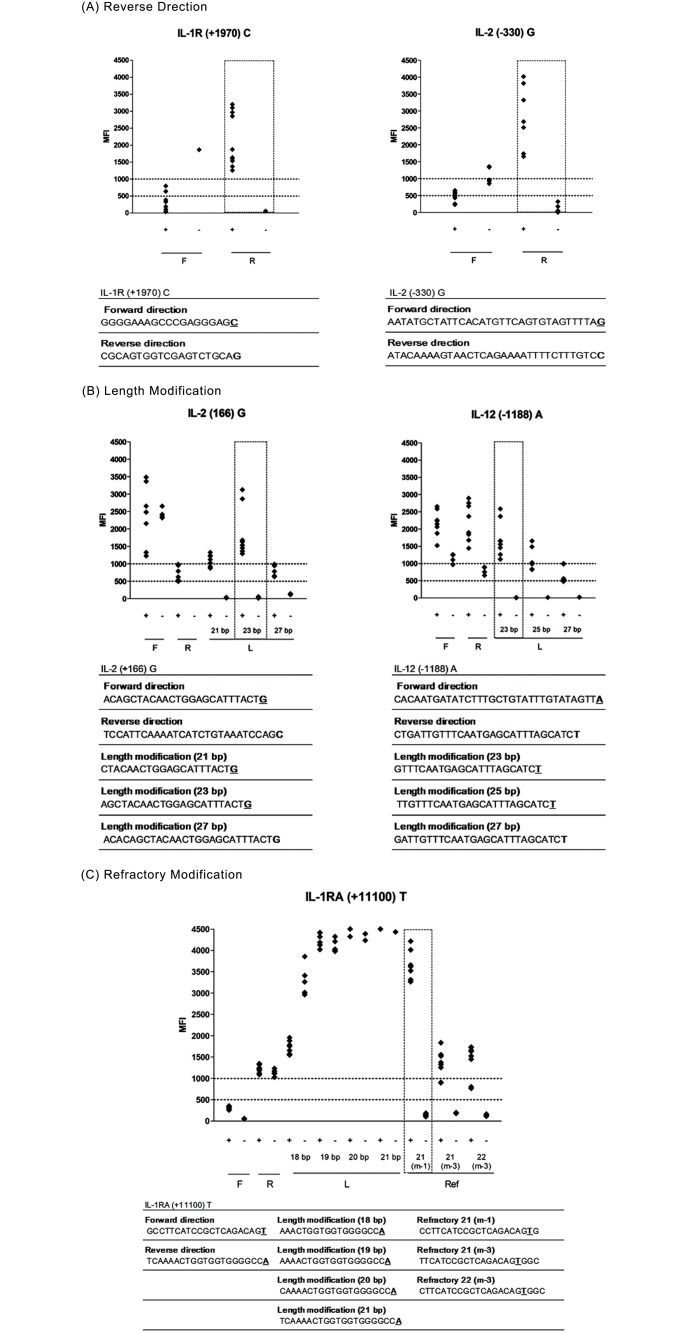
The optimal conditions for high-quality allele-specific primers. Strategy for improving the quality of the primers for IL-2 (-330) G, IL-1R (+1970) C, IL-1β (-511) C, TGF-β1 (codon 25) G and IL-1RA (+11100) T primer. (A) IL-1R (+1970) C and IL-2 (-330) G primers were improved by the reverse modification stage. (B) In the length modification stage, qualities of the IL-1β (-511) C and TGF β1 (codon 25) G primers were improved by adding or removing sequences at the 5′ end of each primer. (C) The IL-1RA (+11100) T primer failed in each of the forward, reverse direction and length modification stages. However, the IL-1RA (+11100) T primer was improved by moving 1 to 3 bp upstream of the SNP region at the allele-specific 3′ end. The sequences were used step by step for optimal extension primer selection. The high-quality primers showed signals higher than 1000 MFI in the positive allele and lower than 500 MFI in the negative allele. F: forward direction, R: reverse direction, L: length modification, Ref: refractory system. The SNP site in each primer is underlined. MFI: median fluorescence intensity.

These primers were between 20–34 bases in length, with melting temperatures of 57.1 ± 1.1°C, and GC contents of 49.5 ± 12.6% (Table 3). The remaining 16 failed primers were 18–29 bases in length, with melting temperatures of 57.5 ± 1.2°C, and GC contents of 55.4 ± 13.4% (false positives; IL-1β (-511) C, IL-10 (-1082) G, IL-10 (-1082) A, IL-12 (-1188) C, IL-12 (-1188) A, TGF-β1 (codon 25) G, TGF-β1 (codon 25) C, TNF-α (-308) A, IFN-γ (5644) A and IFN-γ (5644) T, false negatives; IL-2 (+166) G, IL-6 (-174) G, TGF-β1 (codon 10) C, TGF-β1 (codon 10) T, IL-1RA (+11100) C and IL-1RA (+11100) T). Overall, the reverse direction primers of IL-1R (+1970) C and IL-2 (-330) G demonstrated improved specificity and sensitivity compared to forward primers, as shown in Fig. 3A.

The lengths of the 16 failed primers with forward or reverse direction were further modified from 18 bp to 30 bp. Nine primers (IL-1β (-511) C, IL-2 (166) G, IL-6 (-174) G, IL-10 (-1082) A, IL-12 (-1188) C, IL-12 (-1188) A, TGF-β1 (codon 25) G, TGF-β1 (codon 25) G and TNF-α (-308) A) were selected among a total of 68 primers tested (22–25 bases in length, with melting temperatures of 56.7 ± 6.5°C, and GC contents of 49.6 ± 13.0%). Overall, primers of IL-1β (-511) C and TGF-β1 (codon 25) showed improved positive signals by length modification as seen in Fig. 3B.

In the last assay, the locations of the mismatch SNP sites of these primers were modified to be at 1, 2 or 3 bp upstream of the 3′ end. Three primers (IFN-γ (5644) A, IL-1RA (+11100) C and IL-1RA (+11100) T were selected as high quality among a total 34 primers tested. Overall, the primer of IL-1RA (+11100) T showed improved positive and negative signals by refractory modification as in Fig. 3C.

Finally, four primers (IL-10 (-1082) G, TGF-β1 (codon 10) G, T, IFN-γ (5644) T) did not achieve acceptable quality despite modifications. IL-10 (-1082) G and TGF-β1 (codon 10) extension primers showed false negatives, while the IFN-γ (5644) T primer was showed a false positive result.

### 4. Concordance rate and cytokine SNP frequencies in Korean population

**Fig 4:**
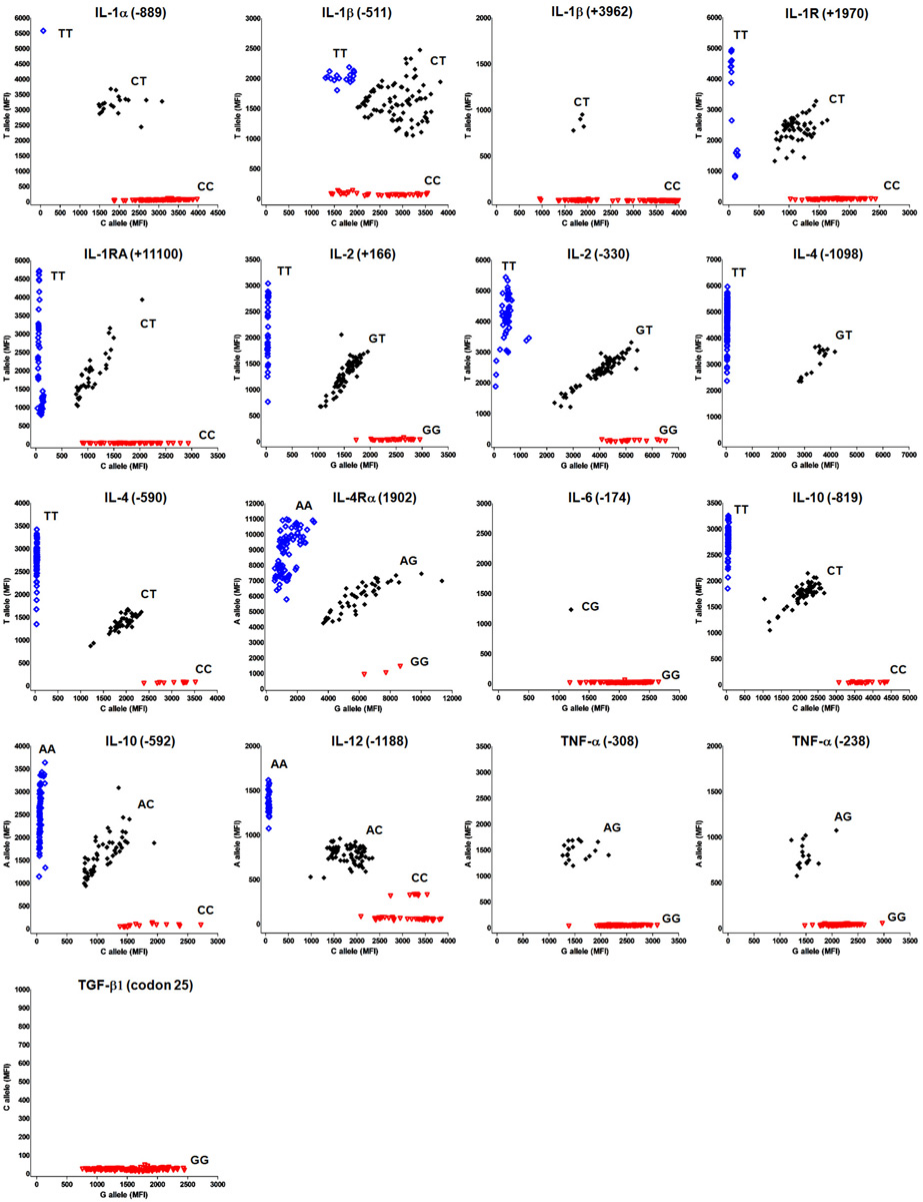
Scatter plot of fluorescence values detected by using allele-specific primer extension (ASPE) on a fluorescence bead array system. Raw fluorescence data are plotted for each cytokine SNP site. The red inverted open triangles represent homozygotes for allele A, the black filled diamonds are heterozygotes and the blue open squares are homozygotes for allele B. MFI: median fluorescent intensity.

After selection of the high-quality primers, seventeen SNPs of 12 cytokine genes were genotyped in 152 healthy Koreans by using the multiplex fluorescence bead array. The genotypes of the SNPs (ex; CC, CT and TT) as determined by the signal intensity ratio between the two corresponding bead types were divided into 3 districts. As a result, IL-1α (-889), IL-1β (-511), IL-1R (+1970), IL-1RA (+11100), IL-2 (+166), IL-2 (-330), IL-4 (-590), IL-4Rα (1902), IL-10 (-592), IL-10 (-819) and IL-12 (-1188) showed all 3 genotypes. IL-1β (+3962), IL-4 (-1098), IL-6 (-174), TNF-α (-238) and TNF-α (-308) were 1 heterozygote and 1 homozygote. TGF-β1 (codon 25) existed only in the homozygous state (GG) (Fig. 4).

In addition, the SNP frequency observed for each cytokine gene by multiplex fluorescence bead array was compared with data identified by sequencing-based typing (SBT). The specificity and sensitivity of the high-quality extension primers identified in a small-scale control panel were examined in 152 normal healthy Koreans; furthermore, the reproducibility and concordance rates were investigated. As a result, very weak positive signals of the six samples that had greater than 500 MFI were excluded from the genotyping results. The comparison results from the bead array method and SBT data in 146 normal healthy Koreans showed 100% concordance rate in IL-1α (-889), IL-1R (+1970), IL-1RA (+11100), IL-2 (+166), IL-4 (-1098), IL-4 (-590), IL-4Rα (1902), IL-6 (-174), IL-10 (-819), IL-10 (-592), IL-12 (-1188), TNF-α (-308), TNF-α (-238) and TGF-β1 (codon 25). These results showed that 14 of 17 SNPs were consistent with the sequencing data (Table 4). The signal values of primers showing 100% concordance rates displayed positive values higher than the average at 2135 MFI, and negative values at less than 500 MFI (Fig. 4).

**Table 4:** Concordance rate of the fluorescence bead array method vs. sequencing-based genotyping.

	SNP	Concordance Rate	
Gene	Position	Count	Rate (%)
IL-1α	-889	146 / 146	100
IL-1β	-511	143 / 146	97.9
	+3962	144 / 146	98.6
IL-1R	+1970	146 / 146	100
IL-1RA	+11100	146 / 146	100
IL-2	+166	146 / 146	100
	-330	143 / 146	97.9
IL-4	-1098	146 / 146	100
	-590	146 / 146	100
IL-4Rα	1902	146 / 146	100
IL-6	-174	146 / 146	100
IL-10	-819	146 / 146	100
	-592	146 / 146	100
IL-12	-1188	146 / 146	100
TNF-α	-308	146 / 146	100
	-238	146 / 146	100
TGF-β1	codon 25	146 / 146	100

IL: interleukin, IFN: interferon, TNF: tumor necrosis factor, TGF: transforming growth factor, R: receptor, RA: receptor antagonist.

On the other hand, three SNPs (IL-1β (-511), IL-1β (+3962), and IL-2 (-330)) were identified as error genotypes. However, two replicate experiments have managed to eliminated these results.

**Fig 5:**
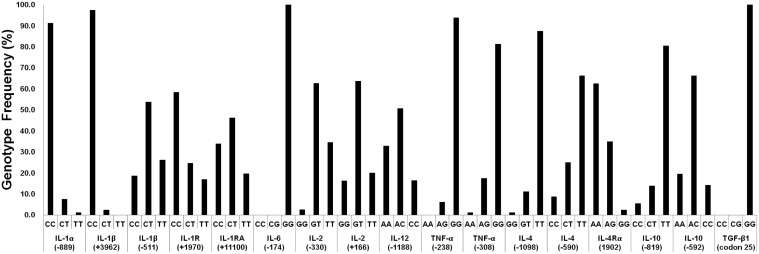
Genotype frequencies of multiple cytokine SNPs in healthy Korean individuals (N = 146). IL: interleukin, IFN: interferon, TNF: tumor necrosis factor, TGF: transforming growth factor, R: receptor, RA: receptor antagonist.

Finally, seventeen SNPs of 12 cytokine genes were genotyped and their frequencies determined in 146 healthy Koreans by the multiplexing system (Fig. 5). For each genotype frequency, an exact test of Hardy-Weinberg proportions was performed among the controls. All genotyped SNPs fit the Hardy-Weinberg equilibrium.

## Discussion

Multiplex PCR is used for pathogen identification [24], high-throughput SNP genotyping [26], mutation analysis [27], gene deletion analysis [28,29], quantitative analysis [30,31], and RNA detection [32,33]. The specificity and efficiency of amplification, as well as the process time for multiplex PCR have been enhanced by various improved primer designs and optimized reaction conditions. However, most multiplex PCR are limited to the amplification of 5 to 10 targets due to primer-primer interactions.

A single-tube 640-plex genotyping method has been reported for the detection of nucleic acid variations on microarrays [25]. According to this method, each specific primer was designed to cover one span between the forward and reverse primers. Among the 13 candidate cytokine genes, 12 cytokine SNPs were successfully defined with the selected allele-specific primers. However, the TGFβ1 gene (codons 10 and 25) was not amplified in the multiplex PCR due to the high GC content of the primer region. The TGFβ1-specific primer was eventually designed to 46 span between the forward and reverse primer, which were successful in the amplification without primer-primer interference. Compared to planar microarrays, suspension arrays benefits from ease of use, low cost, statistical superiority, faster hybridization kinetics, and more flexibility in array preparation. This bead array platform has been utilized in procedures such as allele-specific hybridization [19], single-base extension (SBE) [18,34], allele-specific primer extension (ASPE) [26], and oligonucleotide ligation (OL) assay [35]. Although most manufactured high-density SNP arrays for GWAS generally employ the SBE method, we decided to use the allele-specific primer extension method instead to discriminate between the genotypes of multiple cytokine SNPs. The ASPE assay is in a one-color format and is specifically designed to allow the detection of all SNPs by using two identical extension primers differing only at the 3′ base. One of the extension primers is a perfect match for hybridization to allele A, while the other is the perfect match hybrid for allele B. The ability to detect all SNPs is useful for genotyping custom SNPs [36].

In this study, SNP genotypes were confirmed by scatter plot distribution using Korean control samples. Scatter plots depict median fluorescence intensities for alternate SNP alleles (Fig. 4). To select and improve the extension primers, modifications for the reverse direction, length and refractory were performed (Fig. 2B). Among a total of 204 extension primers tested, 24 primers were selected as high-quality primers in the forward direction step (forward and reverse), and then 12 primers were improved by adjusting the primer modifications (length and refractory). Interestingly, despite the fact that the lengths of 6 pairs of primers were shortened, they were showed a higher signal and increased specificity. These results indicate the significance of primer structures such as hairpin loops. The refractory modification used in this study may be similar to the ARMS technology [31]. However, we chose to move the 3′ end of the SNP region instead of changing the artificial mismatched base pair in the extension primer to improve specificity. As a result, two primers for IFN-γ (5644) A and IL-1RA (+11100) C were improved by moving the third base pair upstream of the SNP region at the allele-specific 3′-end. These results suggest that to improve the specificity of the allele-specific extension primer, the 3′-end of extension primers should be a mismatched site to be located at the second or third base pair rather than the first base pair behind the 3′ end. Also, to validate all the allele-specific extension primers, some genotypes (IL-1β (+3962) TT, IL-6 (-174) CC, TNF-α (-308) AA and TGF-β1 (codon 25) CC, CG) were confirmed to the reference sample from the International Histocompatibility Working Group (IHWG; http://www.ihwg.org/reference/index.html), because the presented genotypes were not detected in the Korean population (result data not shown). However, allele-specific extension primers for IL-4 (-1098) G and TNF-α (-238) A were confirmed only in the heterozygote because IL-4 (-1098) GG and TNF-α (-238) AA genotypes were not detected. In this system, we selected 36 high-quality extension primers of the 40 primers tested. SNP genotypes distinguished by SBT showed the same results with those of multiplex PCR coupled with a fluorescent bead array (Table 4). The frequencies of multiple cytokine SNPs demonstrated in this study were consistent with previous studies also in the Korean population (Fig. 5) [37].

In conclusion, this study indicates that multiplex amplification can be successfully coupled with a fluorescent bead array detection method, and this combined platform provides a fast and high-throughput multiplex detection system for SNPs of multiple cytokine genes in a single tube format. Because cytokines act in complex regulatory networks, and gene—gene interactions are likely to be at the center of many disease associations, simultaneous testing of multiple SNPs are now being introduced to determine such relationships and this should ultimately allow a more accurate estimate of disease risk for individuals with particular cytokine gene profiles.

## References

[1] Gupta S (1988) Cytokines: molecular and biological characteristics. Scandinavian journal of rheumatology Supplement 76: 189–201. 3075076 10.3109/03009748809102969

[2] Bidwell J, Keen L, Gallagher G, Kimberly R, Huizinga T, et al (1999) Cytokine gene polymorphism in human disease: on-line databases. Genes and immunity 1: 3–19. 11197303 10.1038/sj.gene.6363645

[3] Dinarello CA (2007) Historical insights into cytokines. European journal of immunology 37 Suppl 1: S34–45. 17972343 10.1002/eji.200737772 PMC3140102

[4] Mosmann TR, Sad S (1996) The expanding universe of T-cell subsets: Th1, Th2 and more. Immunology today 17: 138–146. 8820272 10.1016/0167-5699(96)80606-2

[5] Noack M, Miossec P (2014) Th17 and regulatory T cell balance in autoimmune and inflammatory diseases. Autoimmunity reviews 13: 668–677. 10.1016/j.autrev.2013.12.004 24418308

[6] Kimura A, Kishimoto T (2010) IL-6: regulator of Treg/Th17 balance. European journal of immunology 40: 1830–1835. 10.1002/eji.201040391 20583029

[7] Ollier WE (2004) Cytokine genes and disease susceptibility. Cytokine 28: 174–178. 15588692 10.1016/j.cyto.2004.07.014

[8] Smith AJ, Humphries SE (2009) Cytokine and cytokine receptor gene polymorphisms and their functionality. Cytokine & growth factor reviews 20: 43–59. 10.1093/ijnp/pyu084 19038572

[9] Meenagh A, Williams F, Ross OA, Patterson C, Gorodezky C, et al (2002) Frequency of cytokine polymorphisms in populations from western Europe, Africa, Asia, the Middle East and South America. Hum Immunol 63: 1055–1061. 12392859 10.1016/s0198-8859(02)00440-8

[10] Shoskes DA, Albakri Q, Thomas K, Cook D (2002) Cytokine polymorphisms in men with chronic prostatitis/chronic pelvic pain syndrome: association with diagnosis and treatment response. The Journal of urology 168: 331–335. 12050565

[11] Dunbar SA (2006) Applications of Luminex xMAP technology for rapid, high-throughput multiplexed nucleic acid detection. Clinica chimica acta; international journal of clinical chemistry 363: 71–82. 16102740 10.1016/j.cccn.2005.06.023 PMC7124242

[12] Johnson VJ, Yucesoy B, Luster MI (2004) Genotyping of single nucleotide polymorphisms in cytokine genes using real-time PCR allelic discrimination technology. Cytokine 27: 135–141. 15304242 10.1016/j.cyto.2004.05.002

[13] Pyo C-W, Hur S-S, Kim Y-K, Choi H-B, Hong Y-S, et al (2003) Polymorphisms of IL-1B, IL-1RN, IL-2, IL-4, IL-6, IL-10, and IFN-γ genes in the Korean population. Human Immunology 64: 979–989. 14522096 10.1016/s0198-8859(03)00173-3

[14] Bagheri M, Abdi-Rad I, Omrani D, Khalkhali HR (2006) Cytokine typing: SNP allele frequencies in the Iranian population. International journal of immunogenetics 33: 193–195. 16712650 10.1111/j.1744-313X.2006.00595.x

[15] Karabon L, Wysoczanska B, Bogunia-Kubik K, Suchnicki K, Lange A (2005) IL-6 and IL-10 promoter gene polymorphisms of patients and donors of allogeneic sibling hematopoietic stem cell transplants associate with the risk of acute graft-versus-host disease. Hum Immunol 66: 700–710. 15993715 10.1016/j.humimm.2005.02.003

[16] Carnt NAW, Hau MD, Garthwaite S, Evans LL, Radford VE, et al (2012) Association of single nucleotide polymorphisms of interleukins-1beta, -6, and-12B with contact lens keratitis susceptibility and severity. Ophthalmology 119: 1320–1327. 10.1016/j.ophtha.2012.01.031 22503230

[17] Otani S, Oda S, Sadahiro T, Nakamura M, Watanabe E, et al (2009) Clinical application of cytokine-related gene polymorphism analysis using a newly developed DNA chip in critically ill patients. Clinical biochemistry 42: 1387–1393. 10.1016/j.clinbiochem.2009.06.005 19527699

[18] Chen J, Iannone MA, Li MS, Taylor JD, Rivers P, et al (2000) A microsphere-based assay for multiplexed single nucleotide polymorphism analysis using single base chain extension. Genome research 10: 549–557. 10779497 10.1101/gr.10.4.549 PMC310857

[19] Armstrong B, Stewart M, Mazumder A (2000) Suspension arrays for high throughput, multiplexed single nucleotide polymorphism genotyping. Cytometry 40: 102–108. 10805929

[20] Iannone MA, Taylor JD, Chen J, Li MS, Ye F, et al (2003) Microsphere-based single nucleotide polymorphism genotyping. Methods in molecular biology (Clifton, NJ) 226: 123–134. 12958493 10.1385/1-59259-384-4:123

[21] Bortolin S (2009) Multiplex genotyping for thrombophilia-associated SNPs by universal bead arrays. Methods in molecular biology (Clifton, NJ) 496: 59–72. 10.1007/978-1-59745-553-4_6 18839105

[22] Hamza IA, Jurzik L, Wilhelm M (2014) Development of a Luminex assay for the simultaneous detection of human enteric viruses in sewage and river water. Journal of virological methods 204: 65–72. 10.1016/j.jviromet.2014.04.002 24747587

[23] Ozaki S, Kato K, Abe Y, Hara H, Kubota H, et al (2014) Analytical performance of newly developed multiplex human papillomavirus genotyping assay using Luminex xMAP technology (Mebgen HPV Kit). Journal of virological methods 204: 73–80. 10.1016/j.jviromet.2014.04.010 24768623

[24] Kim MS, Kang HJ, Park HJ, Yook YJ, Han BD, et al (2011) Development of multiplex PCR method for the analysis of glutathione s-transferase polymorphism. Molecular diagnosis & therapy 15: 285–292. 10.1016/j.bios.2015.01.054 22047155

[25] Krjutskov K, Andreson R, Magi R, Nikopensius T, Khrunin A, et al (2008) Development of a single tube 640-plex genotyping method for detection of nucleic acid variations on microarrays. Nucleic acids research 36: e75 10.1093/nar/gkn357 18539607 PMC2475630

[26] Ye F, Li MS, Taylor JD, Nguyen Q, Colton HM, et al (2001) Fluorescent microsphere-based readout technology for multiplexed human single nucleotide polymorphism analysis and bacterial identification. Human mutation 17: 305–316. 11295829 10.1002/humu.28

[27] Fu G, Miles A, Alphey L (2012) Multiplex detection and SNP genotyping in a single fluorescence channel. PloS one 7: e30340 10.1371/journal.pone.0030340 22272339 PMC3260291

[28] Elnifro EM, Ashshi AM, Cooper RJ, Klapper PE (2000) Multiplex PCR: Optimization and Application in Diagnostic Virology. Clinical Microbiology Reviews 13: 559–570. 11023957 10.1128/cmr.13.4.559-570.2000 PMC88949

[29] Tsuchihashi Z, Dracopoli NC (2002) Progress in high throughput SNP genotyping methods. The pharmacogenomics journal 2: 103–110. 12049172 10.1038/sj.tpj.6500094

[30] Soler S, Rittore C, Touitou I, Philibert L (2011) A comparison of restriction fragment length polymorphism, tetra primer amplification refractory mutation system PCR and unlabeled probe melting analysis for LTA+252 C>T SNP genotyping. Clinica chimica acta; international journal of clinical chemistry 412: 430–434. 10.1016/j.cca.2010.11.012 21094154

[31] Newton CR, Graham A, Heptinstall LE, Powell SJ, Summers C, et al (1989) Analysis of any point mutation in DNA. The amplification refractory mutation system (ARMS). Nucleic acids research 17: 2503–2516. 2785681 10.1093/nar/17.7.2503 PMC317639

[32] Tomiuk S, Hofmann K (2001) Microarray probe selection strategies. Briefings in bioinformatics 2: 329–340. 11808745 10.1093/bib/2.4.329

[33] Bej AK, Mahbubani MH, Miller R, DiCesare JL, Haff L, et al (1990) Multiplex PCR amplification and immobilized capture probes for detection of bacterial pathogens and indicators in water. Molecular and cellular probes 4: 353–365. 2280781 10.1016/0890-8508(90)90026-v

[34] Cai H, White PS, Torney D, Deshpande A, Wang Z, et al (2000) Flow cytometry-based minisequencing: a new platform for high-throughput single-nucleotide polymorphism scoring. Genomics 66: 135–143. 10860658 10.1006/geno.2000.6218

[35] Iannone MA, Taylor JD, Chen J, Li MS, Rivers P, et al (2000) Multiplexed single nucleotide polymorphism genotyping by oligonucleotide ligation and flow cytometry. Cytometry 39: 131–140. 10679731

[36] Steemers FJ, Gunderson KL (2007) Whole genome genotyping technologies on the BeadArray platform. Biotechnology journal 2: 41–49. 17225249 10.1002/biot.200600213

[37] Gonzalez-Galarza FF, Christmas S, Middleton D, Jones AR (2011) Allele frequency net: a database and online repository for immune gene frequencies in worldwide populations. Nucleic acids research 39: D913–919. 10.1093/nar/gkq1128 21062830 PMC3013710

